# Editorial: Fetal-Maternal Immune Interactions in Pregnancy

**DOI:** 10.3389/fimmu.2019.02729

**Published:** 2019-11-27

**Authors:** Nandor Gabor Than, Sinuhe Hahn, Simona W. Rossi, Julia Szekeres-Bartho

**Affiliations:** ^1^Institute of Enzymology, Research Centre for Natural Sciences, Budapest, Hungary; ^2^Maternity Private Clinic of Obstetrics and Gynaecology, Budapest, Hungary; ^3^First Department of Pathology and Experimental Cancer Research, Semmelweis University, Budapest, Hungary; ^4^Department of Biomedicine, University Hospital Basel, Basel, Switzerland; ^5^Department of Medical Biology, Medical School, University of Pécs, Pécs, Hungary; ^6^MTA - PTE Human Reproduction Research Group, Pécs, Hungary; ^7^János Szentágothai Research Centre, Medical School, University of Pécs, Pécs, Hungary; ^8^Endocrine Studies, Centre of Excellence, Pécs, Hungary

**Keywords:** infection, inflammation, miscarriage, obstetrical syndromes, placenta, pregnancy, maternal-fetal immune tolerance

While contemplating the best way to visually present our Research Topic, the “Tree of Life” concept emerged. Indeed, the developing fetus, attached to the placenta, resembles a tree with its roots. The different cell types, molecules, and their interactions in the placenta that help to nurture, maintain and protect the new life are all important factors in creating a safe environment for the semi-allograft fetus.

Human placentation is unique, as it allows an intimate contact between maternal and fetal cells at the maternal-fetal interface throughout pregnancy, which results in tightly controlled immune interactions between the mother and her child.

Paternal antigens expressed by the fetus are recognized as foreign, which leads to the activation of the maternal immune system. At the same time, several regulatory mechanisms are activated, to create a favorable immunological environment for the developing fetus. The former include altered antigen presentation and T cell differentiation, proliferation and activation of regulatory cells, as well as the action of hormones, cytokines and other soluble factors.

With this Research Topic we have recognized the need to discuss the role of immunological mechanisms, microvesicular and molecular transport of biological information and signaling between the mother and the fetus in promoting immunological tolerance. We have also focused on the effects of maternal infections and local or systemic inflammation that may lead to the failure of these tolerance mechanisms and the development of a spectrum of pregnancy complications, which have an impact on placental and fetal development and health later in life.

**Dendritic cells** (DC) have a critical role in deciding, whether foreign antigens are to be considered as dangerous or neutral and consequently in the acceptance or rejection of the foreign fetal antigens by the maternal immune system. Ehrentraut et al. report the gestational age-dependent differential regulation of peripheral blood dendritic cell subsets during normal pregnancy. Miscarriage is associated with dysregulations in the myeloid peripheral blood dendritic cell subsets, together with lower regulatory T cell frequencies.

By down regulating the immune response, antigen specific **regulatory T cells** play an important role in controlling anti-fetal immune reactions. Antigen-specific regulatory T cells are crucial for the establishment of immunological tolerance. While self-reactive Treg cells contribute to the maintenance of self-tolerance, paternal antigen-specific T cells control the immune response to paternal antigen expressing cells. Tsuda et al. review the role of regulatory T cells in establishing an appropriate immunological relationship between the mother and the fetus. Paternal antigen-specific Treg cells accumulate in the murine placenta, and recent studies have identified these cells at the feto-maternal interface of humans. Abnormal expression or function of antigen specific Treg cells has been observed in miscarriage and preeclampsia. Indeed, in an original research article by the same authors, Tsuda et al. shows that the number of clonally expanded decidual effector regulatory T cells increases in late gestation, but not in preeclampsia.

Kieffer et al. discuss the role of memory T cells in the establishment of tolerance toward allogeneic paternal antigens and their important role in inducing fetal tolerance.

Because inflammation is implicated as a causal factor in preeclampsia and because Treg cells are able to control inflammation, the paper of Robertson et al. discusses the potential therapeutic use of Treg cells. Several possibilities—including pharmacological interventions to target Treg cells and *in vitro* Treg cell generation—are considered, as possible new approaches in the therapy of inflammatory conditions.

Tolerance to antigens are also matter of discussion in the review of Hahn et al. where the role of feto-maternal chimerism is examined in relation to the development of preeclampsia, or later in life, of autoimmune diseases.

**Other cellular immunological players** are largely responsible for the balance between tolerance and inflammation throughout pregnancy. As elaborated by Vacca et al., decidual innate lymphoid cells (ILCs) (that include NK cells, ILC3, and ILC1), may play a key role in the establishment and maintenance of pregnancy, orchestrating both the tolerogenic and the inflammatory phases, by interacting with stromal cells, neutrophils, myelomonocytic cells, and T lymphocytes.

Köstlin-Gille et al. present novel data about the role of HIF-1α production in myeloid-derived suppressor cells. They show that abrogation of HIF-1α expression in this population results in increased abortion rates in mice.

The review from Reyes and Golos discusses the duplicitous nature of Hofbauer cells. These villous macrophages with M2-like profile play a role in placental development; however, they may produce pro-inflammatory cytokines and mediators that damage the villous cell barrier. Hofbauer cells are ineffective in controlling most TORCH infections, while contributing to vertical transmission of pathogens by harboring them as placental reservoirs.

The paper by Pollheimer et al. reviews how **invasive extravillous trophoblasts (EVTs)** develop and migrate into the uterus where these fetal cells remodel maternal spiral arteries, a process critical for adapting blood flow and nutrient transport to the developing fetus. In the decidua, EVTs encounter various maternal cells including decidual macrophages and uNK cells, which regulate EVT functions by growth factors and cytokines. Failures in this mechanism provides the basis for pregnancy complications such as preeclampsia or recurrent abortion.

Among **proteins involved in modulation of immune responses** during pregnancy, several act as checkpoint molecules. CTLA-4 (Cytotoxic T-lymphocyte-associated protein 4), PD-1 (Programmed Cell Death 1), and TIM-3 (T Cell Immunoglobulin Mucin 3), play key roles in immune defense against infections, prevention of autoimmunity, and tumor immune evasion. Here, Miko et al. review the involvement of these molecules in immuno-regulatory processes during normal pregnancy and in pregnancy complications.

The inhibitory ligand of PD-1, PD-L1 (Programmed Cell Death 1 Ligand 1) has a critical role in the induction and maintenance of immune tolerance to self by modulating the activation threshold of T-cells and limiting T-cell effector responses. Okuyama et al. report on higher levels of soluble PD-L1 in the serum of pregnant women compared to non-pregnant women. The results of functional assays support that sPD-L1 may exert immunoregulatory functions during pregnancy.

Galectin-9 (the inhibitory ligand of TIM-3) belongs to the large immunomodulatory family of galectins, reviewed by Blois et al. Galectins constitute a phylogenetically conserved family of soluble β-galactoside binding proteins, which contribute to placentation by regulating trophoblast development, migration and invasion, angiogenesis, and maternal tolerance to the semi-allogeneic fetus. The altered expression of galectins is associated with infertility and pregnancy complications, and their potential therapeutic role in pregnancy complications is also suggested.

Balogh et al. investigated placenta-specific members of this galectin family, which are expressed by a gene cluster on Chromosome 19 that emerged in anthropoid primates. Gal-13 and gal-14 are predominantly expressed by the syncytiotrophoblast at the lining of the maternal-fetal interface, and their expression is down-regulated in miscarriages. Gal-13 and gal-14 bind to T cells, where they inhibit activation, induce apoptosis, and enhance IL-8 production, suggesting that these galectins are key players in regulating the maternal adaptive immune response.

**Progesterone** is indispensable for both the establishment and maintenance of pregnancy in most mammals. As shown by Shah et al. the immune system is increasingly activated during pregnancy, which is counterbalanced by a tolerant immune environment (IL-10 and regulatory-T cells) that gradually reverses prior to the onset of labor. Progesterone suppresses while progesterone receptor blockers enhance the release of inflammatory cytokines and cytotoxic molecules by antigen-specific CD4 and CD8 T cells. Furthermore, progesterone regulates the sensitivity of differentiated memory T cells to antigen stimulation.

Progesterone and a progesterone induced protein, PIBF, are important players in re-adjusting the functioning of the maternal immune system during pregnancy. Szekeres-Bartho et al. review the role of PIBF (carried by extracellular vesicles) in embryo-maternal immune-interactions. PIBF mediates the immunological actions of progesterone. By upregulating Th2 type cytokine production and by down-regulating NK activity, PIBF contributes to the altered attitude of the maternal immune system. Aberrant production of PIBF isoforms results in the loss of immuno-regulatory functions, and pregnancy failure. They also show pre-implantation embryos produce EVs both *in vitro*, and *in vivo*. PIBF transported by the EVs from the embryo to maternal lymphocytes induces increased IL-10 production by the latter, this way contributing to the Th2 dominant immune responses.

Littauer and Skountzou review several studies that demonstrated the vulnerability of pregnant women to infectious diseases, concluding that modulation of inflammation by pregnancy hormones might be the reason.

**Other immunomodulatory molecules** include miRNAs that control inflammation and tolerance in pregnancy as reviewed by Kamity et al. The paradigm of a sterile intrauterine microenvironment was challenged by the detection of microflora in gestational tissues and amniotic fluid in the absence of inflammation. Therefore, adaptation to microbial products may be critical for the prevention of excessive maternal inflammatory responses and fetal rejection. The presented model herein suggests that repeated exposures to microbial products induce a tolerant phenotype at the maternal-fetal interface mediated by specific miRNAs mostly contained within placental EVs, and that the impairment of this mechanism will result in pathological inflammatory responses contributing to pregnancy complications.

Original research by Twisselmann et al. presents that IgG Fc glycosylation patterns of infants depend on their gestational ages. Preterm infants acquire reduced amounts of IgG via trans-placental transfer which might explain their high susceptibility for infections. Moreover, there is a qualitative shift in the type of IgG Fc glycosylation toward a pro-inflammatory pattern in preterm infants that might contribute to their increased risk for chronic inflammatory diseases such as bronchopulmonary dysplasia.

Immune tolerance toward paternal and fetal antigens is crucial for reproductive success and the breakdown of this mechanism is implicated in the pathophysiology of **pregnancy complications**, including miscarriage, preterm birth and preeclampsia.

van der Zwan et al. show that pregnancy outcomes can be influenced by the presence of allo-reactive HLA-C CD8+ T cells originating from viral memory response (e.g., Influenza, Epstein-Barr virus, Cytomegalovirus, Varicella).

The review paper by Schepanski et al. focuses on the effect of the maternal immune environment on fetal brain development. Vertical transmission of hormones, maternal immune cells and cytokines might affect brain development as well as cognitive and intellectual performances of the offspring. Recent data underpin that brain development in response to prenatal stress challenges can be altered across several generations, independent of a genetic predisposition, supporting an epigenetic inheritance.

Spontaneous preterm birth is the leading cause of new-born deaths; therefore, there is a huge unmet clinical need for its prevention. In a cross-study meta-analysis, Vora et al. evaluate genome-wide differential gene expression signals in maternal and cord blood taken from women with preterm or term births, and identified genes differentially expressed in preterm birth. These were enriched in immune-related pathways, showing up-regulation of innate immunity and down-regulation of adaptive immunity. Several genes were differentially expressed at mid-gestation, suggesting their potential clinical utility as biomarkers.

Preeclampsia is one of the deadliest obstetrical syndrome. The placenta has a key role in the pathogenesis of preeclampsia, characterized by maternal systemic inflammation, which may be triggered by distinct underlying mechanisms in early pregnancy. Immunological incompatibility between the mother and the fetus is strongly indicated, and genetic factors linking immunological pathways to preeclampsia predisposition have been identified. In a mini-review, Lokki et al. discuss genetic variations in immunological factors in the context of preeclampsia and explore immunogenetic and immunomodulary mechanisms contributing to loss the of tolerance, inflammation, and autoimmunity that may lead to preeclampsia.

In a review, Geldenhuys et al. summarize cellular and molecular background of normal placentation and give details on how placentation is disrupted in the “placental subtype” of preeclampsia as a result of failure of tolerance or infections. These lead to aberrant activation of innate immune cells and imbalanced differentiation of T-helper cell subsets, creating a cytotoxic environment *in utero*, placental developmental problems, and eventually excessive maternal systemic inflammation.

In a complex systems biology study, Than et al. integrated different “omics,” clinical, placental, and functional data to gain insights into the early molecular pathways of preeclampsia. Distinct maternal and placental disease pathways were revealed to interact and influence the clinical presentation. As a paradigm shift, the activation of maternal disease pathways, including inflammatory changes, was detected upstream of placental dysfunction, and placental disease pathways to be superimposed on these maternal pathways. This warrants for the central pathologic role of pre-existing maternal diseases or perturbed maternal-fetal-placental immune interactions. The description of these novel pathways in the “molecular phase” of preeclampsia and the discovery of new biomarkers by this study may enable the early identification of patients with distinct molecular preeclampsia phenotypes.

## Author Contributions

All authors listed have made a substantial, direct and intellectual contribution to the work, and approved it for publication. All authors have contributed equally to this work.

### Conflict of Interest

The authors declare that the research was conducted in the absence of any commercial or financial relationships that could be construed as a potential conflict of interest.

